# Isolation and characterization of cells derived from human epithelial rests of Malassez

**DOI:** 10.1007/s10266-018-0397-7

**Published:** 2018-11-26

**Authors:** Kayoko Kitajima, Ridhima Das, Xiao Liang, Evelyn Neppelberg, Anne Christine Johannessen, Daniela Elena Costea, Masaru Igarashi

**Affiliations:** 1 Department of Endodontics, The Nippon Dental University School of Life Dentistry at Niigata, 1-8 Hamaura-cho, Chuo-ku, Niigata, 951-8580 Japan; 2 Center for Cancer Biomarkers CCBIO and Gade Laboratory for Pathology, Department of Clinical Medicine, University of Bergen, N-5021 Bergen, Norway; 3 Section of Neurology, Department of Clinical Medicine, Faculty of Medicine, University of Bergen, N-5021 Bergen, Norway; 4 Head and Neck Clinic, Haukeland University Hospital, N-5021 Bergen, Norway; 5 Department of Pathology, Haukeland University Hospital, N-5021 Bergen, Norway

**Keywords:** Rests of Malassez, Periodontal ligament, Immunohistochemistry, Human, Oral mucosa

## Abstract

The epithelial rests of Malassez (ERMs) might represent a valuable source of oral epithelial cells with stem cell properties. The purpose of this study was to isolate and characterize cells derived from human ERM, and compare them with cells derived from matched normal oral mucosa (NOM). Matched tissue specimens of the periodontal ligament of extracted tooth and NOM were collected. Cells were isolated in culture, then characterized by immunohistochemistry and flow cytometry for expression of pancytokeratin, ESA, PDGFRB, CD31 and CD44. 3D organotypic cultures were constructed by growing epithelial cells on top of fibroblast-populated collagen gels. Both ERM and NOM-isolated cells expressed the markers of epithelial lineage (ESA and pancytokeratin), and to some extent PDGFR, an indicator of a more mesenchymal phenotype, but not the endothelial cell marker CD31. Cells with epithelial morphology were isolated from periodontium of cervical, middle and apical parts of the root, but contained a significantly lower percentage of ESA and pancytokeratin-positive cells than when isolating cells from NOM (*p* < 0.001). ERM cells expressed a significantly higher percentage of the stem cell-related molecule CD44 (cervical 92.93 ± 0.25%, middle 93.8 ± 0.26%, apical 94.36 ± 0.41%) than cells isolated from NOM (27.8 ± 1.47%, *p* < 0.001). When grown in 3D organotypic cultures and in collagen gels, ERM cells formed a less differentiated epithelium than NOM cells, but expressing pancytokeratin and vimentin. In conclusion, epithelial cells could be isolated from human periodontium and grown in culture; their in vitro characterization indicates that they have a less differentiated phenotype compared with cells derived from normal oral epithelium.

## Introduction

Hertwig’s epithelial sheath is the origin of the epithelial rests of Malassez (ERMs) and contributes to the growth of roots. ERMs are considered to participate in the development of radicular cysts. Hertwig’s epithelial sheath and ERMs thus play important roles, in both physiological and pathological root-related processes, but their characterization of and participation in these processes are not yet known.

Mutual epithelial–mesenchymal interactions are thought to play an important role in tooth growth and morphogenesis. When tooth root formation starts, the internal and external enamel epithelium, which have completed crown formation, bend at the tooth cervix and extend its epithelial tip to differentiate into Hertwig’s epithelial sheath, which separates the dental papilla and dental sac, resulting in the formation of the tooth root [1, 2].

Once tooth root formation is complete, Hertwig’s epithelial sheath contracts, but a part of it remains in the periodontal ligament space as ERMs. Orban and Weinmann [3] reported that ERMs near the tooth cervix bind with the junctional epithelium and convert to pocket epithelium. It also appears that ERMs that are normally in a static state start to proliferate and form the epithelial lining of radicular cysts when infectious antigenic substances resulting from dental pulp diseases are discharged from the root canal through the apical foramen [4, 5]. Apical periodontal diseases include suppurative apical periodontitis and chronic inflammation, the latter of which may be classified into periapical granuloma or radicular cyst. Clinically, radicular cysts are more likely to be refractory than the former two conditions, which may be related to the fact that the inner wall of the cyst is lined with epithelium.

There are many unanswered questions, such as why do ERMs remain around the tooth apex after completing their role in root formation, and what is the mechanism of the switch from the quiet, normal state, to the proliferative state leading to the formation of epithelium on radicular cysts as a result of stimulation?

We hypothesized that our understanding of the mechanisms of radicular cyst development will advance by isolating cells from ERMs and learning about their characteristics.

The purpose of this study was to characterize the cells derived from human ERM and compare them with cells derived from matched normal oral mucosa (NOM).

## Materials and methods

### Tissue specimens and primary cell isolation

Matched tissue specimens from the periodontal ligament (PDL) of extracted tooth and NOM were collected after informed consent from healthy patients undergoing wisdom tooth extraction (*N* = 3). Cells were isolated in culture following the standard explant method at 37 °C in 5% CO_2_, in a humidified incubator. Cells were used from the second or third passage.

Extracted teeth and NOM samples were transported on ice in transport medium: Dulbecco’s modified Eagle’s medium (DMEM) (Sigma, St. Louis, MO, USA) with 2% antibiotics–antimycotics (GibcoBRL, Grand Island, NY, USA). After transport, the extracted teeth and biopsy were washed twice, 5 min each time, with fresh transport medium.

The PDL attached to the cervical, middle and apex one-third of the root was removed with a scalpel and collected separately under a dissecting microscope. PDL and NOM tissues were cut in approximately 1 mm^3^ pieces, allowed to adhere to cell culture dishes (Nunclon™ Delta, Thermo Fisher Scientific, CA, US) by letting them for 3–5 min to air dry opened in the sterile hood. After that, culture medium was gently added to the dish, avoiding detachment of the tissue explants. The culture medium used was FAD-FBS medium: DMEM/HAM’s F12: 3/1 with 0.4 µg/ml hydrocortisone, 5 µg/ml insulin, 20 µg/ml transferrin, 50 µg/ml L-ascorbic acid (all from Sigma). Mitomycine C (Sigma)-inactivated 3T3 fibroblasts (10–100 µl/ml of mitomycin C solution per milliliter of culture medium for 2 h) were added to the dishes planed for isolation of epithelial cells and incubated in keratinocyte serum-free media (KSFM, GibcoBRL) supplemented with 1 ng/ml epidermal growth factor (EGF human recombinant), 25 µg/ml bovine pituitary extract (BPE), 20 µg/ml l-glutamine, 1% AB/AM (100 U/ml penicillin, 100 µg/ml streptomycin, and 0.25 µg/ml amphotericin B) (all from GibcoBRL). The dishes planed for isolation of fibroblasts were incubated in fibroblast-specific medium (DMEM supplemented with 10% FBS, Sigma, 20 µg/ml l-glutamine, and 1% AB/AM). Outgrowths of cells from tissue explants were morphologically assessed. Despite incubation in lineage-specific medium, some outgrowths of the other cell type could be observed sporadically in the dishes. For proper separation, the epithelial- or fibroblast-looking outgrowths were separately detached from dishes using plastic cloning rings (Sigma) attached with Vaseline (Sigma) on the dishes around individual explants with a specific cellular morphology. Trypsin 10× (Sigma) was added inside the clonal rings, and the cells surrounding an explant with a uniform morphology of either epithelial, or fibroblastic phenotype were detached. Cells with the same morphology from different explants were then pooled together to eliminate the risk of clonality of isolated cells and farther propagated in lineage-specific medium.

All cells were used in their third to fourth passage (split ratio of 1:4), at a viability more than 80%, kept in a humidified atmosphere with 5% CO_2_ at 37 °C.

### Immunohistochemistry and flow cytometry

Cells were grown on 16 mm^2^ cover-slips in 12-well plates, in their respective growth medium. After 5 days, cells were fixed in 4% formalin for 20 min at room temperature (RT) and kept in PBS at 4 °C until used. Antibody against pancytokeratin (DAKO, Glostrup, Denmark) was used for 1 h. Afterwards, the Envision + system-HRP stain system (DAKO) was used following manufacturer’s instructions, for 30 min. The presence of antigen was visualized with DAB (3,3′diaminobenzidine, DAKO) for 10 min. The slides were counterstained with haematoxylin (DAKO), dehydrated through an ascending graded series of alcohol, xylene and then mounted with an alcohol soluble mounting medium (Eukit, DAKO). Sections treated with antibody diluent instead of primary antibody were used as negative controls. For staining of 3D organotypic cultures and gels, 3 µm sections were cut, deparaffinized and rehydrated by immersion in xylene and diminishing concentrations of alcohol. Retrieval of the epitope was performed by heating the sections in a microwave oven in a pH 6.0 target retrieval buffer (DAKO). For pancytokeratin staining, sections were incubated with 1× proteinase K for 10 mins at room temperature. Endogenous enzyme activity and unspecific binding were blocked using peroxidase block and 10% normal goat serum respectively (both from DAKO). Sections were then incubated over night at 4 °C with one of the following monoclonal mouse anti-human primary antibodies: anti-pancytokeratin (1:2000, DAKO), and anti-vimentin (1:2000, DAKO). Envision+^®^ anti-mouse (DAKO) was used to detect the site of reaction according to the manufacturer`s instructions. And, the reaction was visualized using 3,3′-diaminobenzidine tetrahydrochloride (DAB). Incubation with primary antibody was omitted for negative control sections, and normal human oral mucosa samples have been used as a positive control. Sections were then counterstained with hematoxylin (DAKO), dehydrated and cover-slipped.

For fluorescent activated cell sorting (FACS), cells were detached using trypsin-EDTA 2.5% (Sigma), then stained with the following antibodies: anti-ESA-APC conjugated (Biomed, USA), anti-PDGFRB-PE conjugated (CD140b-PE conjugated, R&D Systems, UK), anti-CD44-PE (R&D Systems, USA), anti-CD31-PE conjugated (R&D Systems, USA), and isotype control IgG2ak-PE and IgG2ak-APC (R&D Systems, USA) at 1:100 dilution in phosphate-buffered saline (PBS) (Invitrogen). DAPI nuclear dye (Sigma) was used at 1 µg/ml to exclude dead cells. All analyses were performed on the FACS aria SORP (Becton Dickinson, USA).

### 3D assays

3D organotypic cultures were obtained by growing epithelial cells on top of fibroblast-populated collagen type I (BD Biosciences) biomatrices, using a protocol well-established in our laboratory [6]. The organotypic cultures were grown in serum-free FAD medium without addition of EGF. The cultures were lifted at air–liquid interface at day 4 and harvested after 10 days, formalin fixed and paraffin embedded or fresh frozen, as previously described.

The epithelial cells were also incubated in collagen gels, formalin fixed, paraffin embedded and sectioned. These 3D sections were stained hematoxylin and eosin.

### Statistical analysis

Data were presented as mean ± SD. One-way Anova was used to compare the expression of different markers in the isolated cells. At least three repeats were performed. All statistical analyses were performed using the statistical package IBM SPSS version 15 (IBM, USA). *p* values less than 0.01 were considered statistically significant.

## Results

**Fig. 1:**
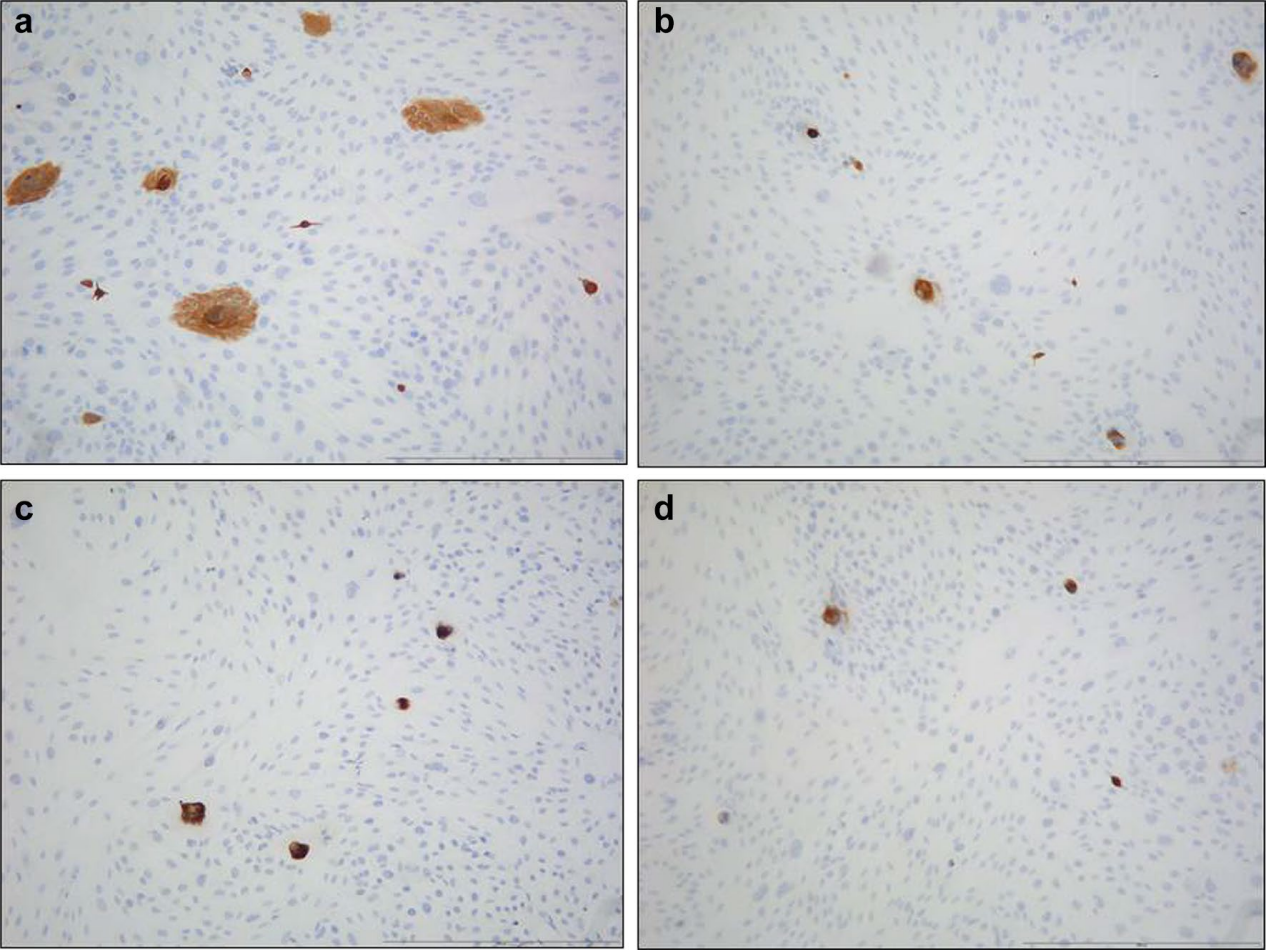
Pancytokeratin staining of cells isolated from NOM and ERM grown in monolayer. **a** Primary gingival keratinocytes from NOM. **b** Primary cells isolated from ERM at cervical part of the root(REM-C). **c** Primary cells isolated from ERM at middle part of the root(REM-M). **d** Primary cells isolated from ERM at apical part of the root (REM-A) (original magnification × 100, scale bar 100 µm). Cells with epithelial morphology and expressing pancytokeratin could be isolated from both ERM and NOM periodontium. However, the number of pancytokeratin-positive cells isolated from PDL at all root levels was very low, significantly lower than when isolating cells from NOM (*p* < 0.001)

**Fig. 2:**
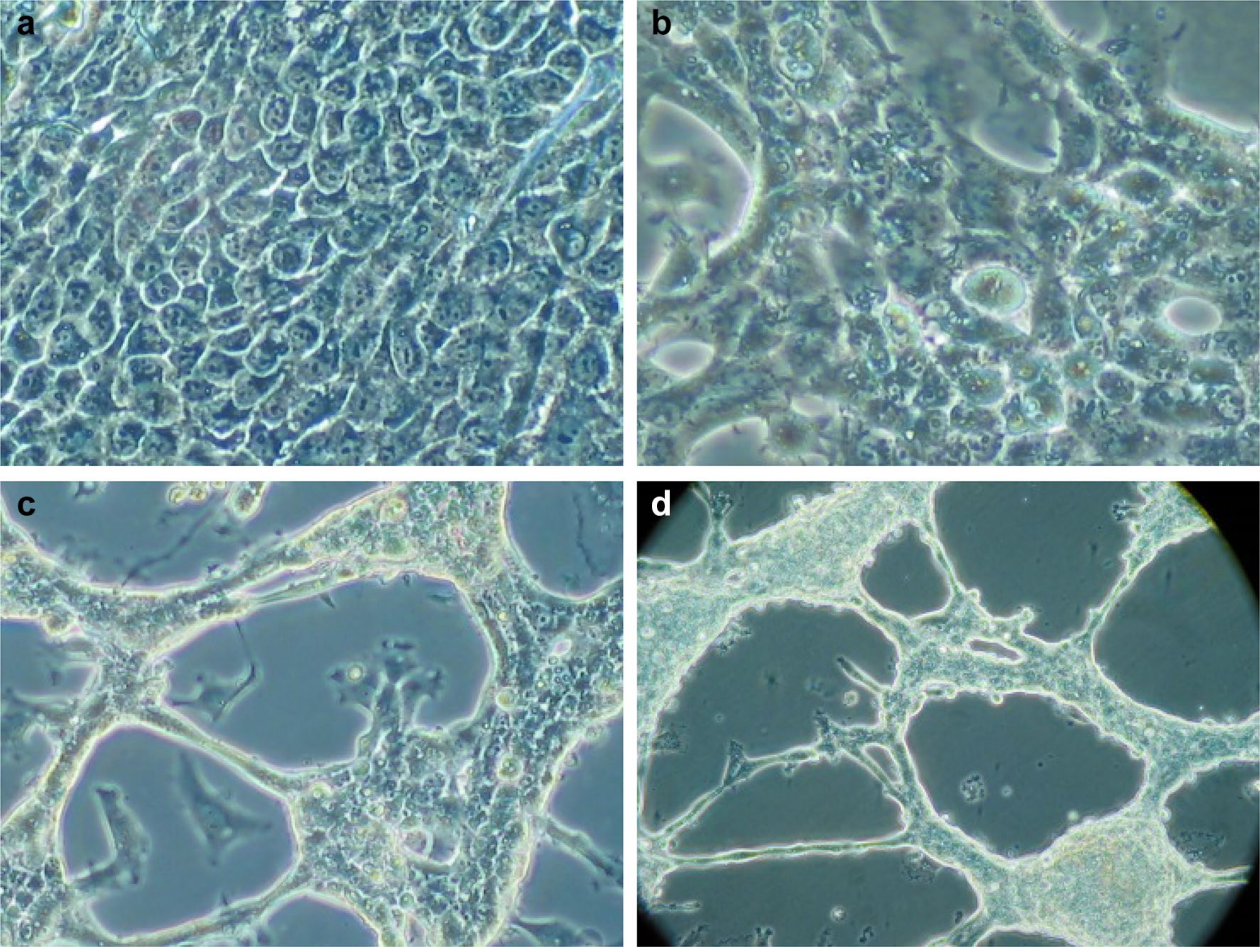
The pattern of growth in culture from human NOM and ERM grown in monolayer. **a** Primary gingival keratinocytes from NOM. **b** Primary cells isolated from ERM-C. **c** Primary cells isolated from ERM-M. **d** Primary cells isolated from ERM-A. The pattern of growth in culture was also different, with ERM cells forming a network of cellular strands while NOM cells formed a uniform, continuous sheet of monolayer cells (original magnification × 400 for **a** and **b**, × 200 for **c** and × 100 for **d**)

Cells with epithelial morphology and expressing pancytokeratin could be isolated (with a similar success rate) from periodontium of cervical (REM-C), middle (REM-M) and apical (REM-A) parts of the root (Fig. 1). However, the number of pancytokeratin-positive cells isolated from PDL at all root levels was very low, significantly lower than when isolating cells from NOM (*p* < 0.001) (Fig. 1).The pattern of growth in culture was also different, with ERM cells forming a network of cellular strands while NOM cells formed a uniform, continuous sheet of monolayer cells (Fig. 2).

**Fig. 3:**
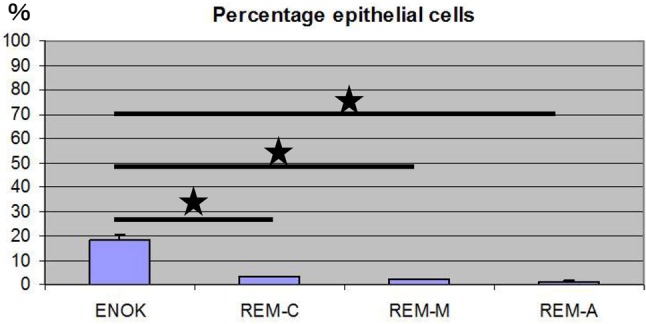
Percentage of epithelial cells (ESA positive cells) by flow cytometry. Both ERM and NOM(ENOK) cells expressed the markers of epithelial lineage ESA. The statistical significant difference was accepted between NOM and REM-C, NOM and REM-M and NOM and REM-A

**Fig. 4:**
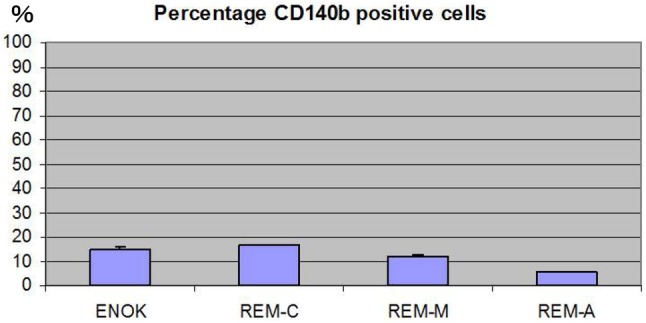
Percentage of PDGFR positive cells by flow cytometry. Both ERM and NOM(ENOK) cells expressed to some extend PDGFR (CD140b), an indicator of a more mesenchymal phenotype. There was no significant difference in each cell which appeared to be statistical

**Fig. 5:**
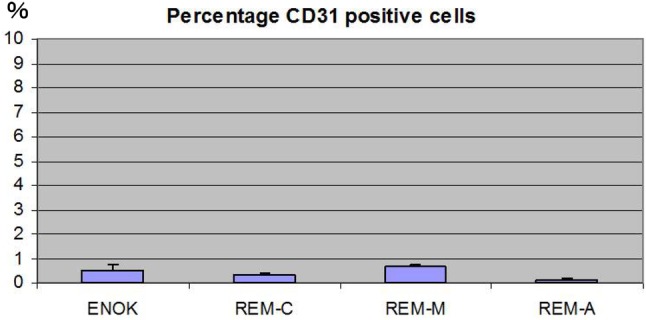
Percentage of CD31 positive cells by flow cytometry. ERM and NOM(ENOK) cells did not express the endothelial cell marker CD31 so much. There was no significant difference in each cell which appeared to be statistical

**Fig. 6:**
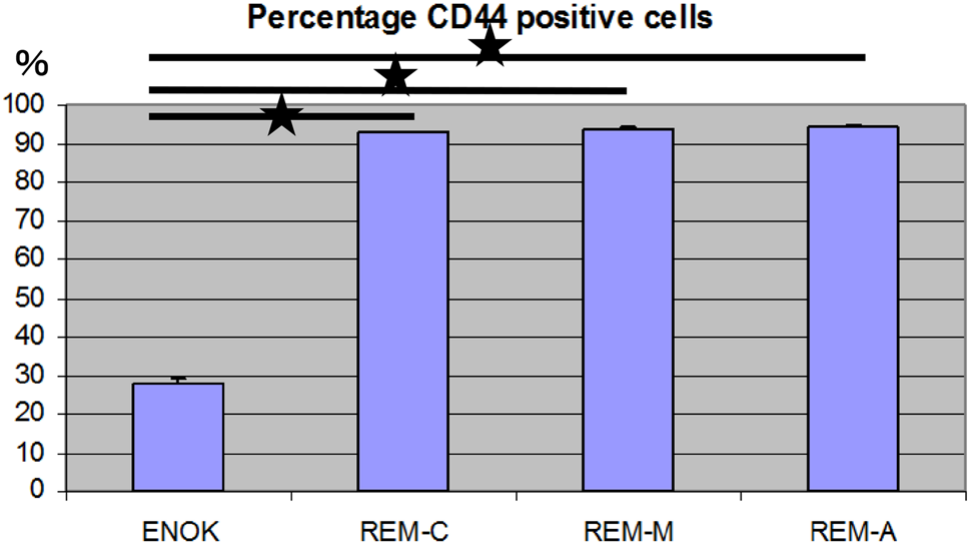
Percentage of CD44 positive cells by flow cytometry. ERM cells expressed a significantly higher percentage of the stem cell-related adhesion molecule CD44 (cervical 92.93 ± 0.25%, middle 93.8 ± 0.26%, apical 94.36 ± 0.41%) than cells isolated from NOM (27.8 ± 1.47%, *p* < 0.001). The statistical significant difference was accepted between NOM and REM-C, NOM and REM-M and NOM and REM-A

Both ERM and NOM cells expressed the markers of epithelial lineage ESA (Fig. 3) and pancytokeratin (Fig. 1), and to some extent PDGFR (CD140b), an indicator of a more mesenchymal phenotype (Fig. 4), but not the endothelial cell marker CD31 (Fig. 5). ERM cells expressed a significantly higher percentage of the stem cell-related adhesion molecule CD44 (cervical 92.93 ± 0.25%, middle 93.8 ± 0.26%, apical 94.36 ± 0.41%) than cells isolated from NOM (27.8 ± 1.47%, *p* < 0.001) (Fig. 6).

**Fig. 7:**
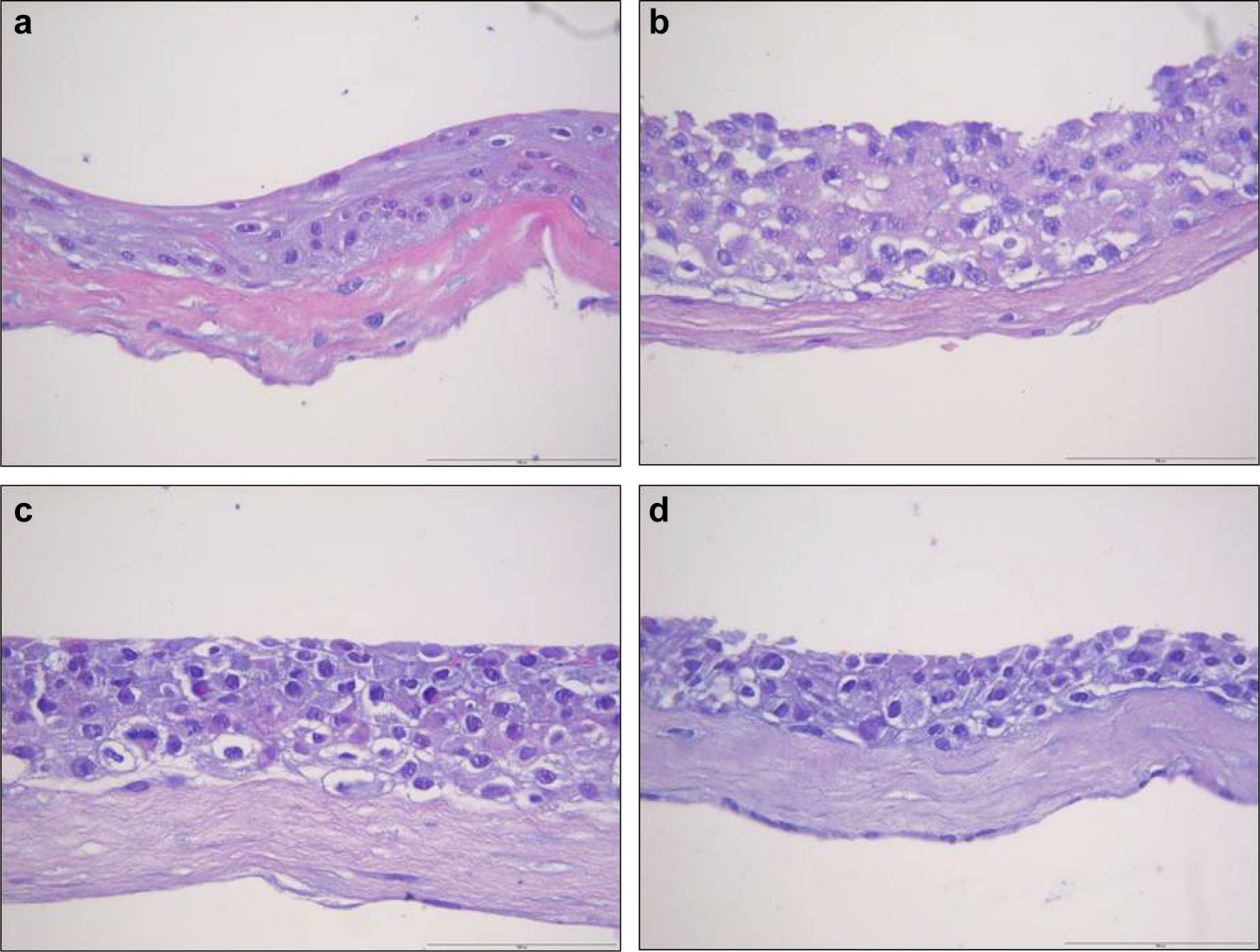
NOM and ERM cells grown in 3D organotypic culture. **a** NOM. **b** REM-C. **c** REM-M. **d** REM-A (original magnification × 200, scale bar 100 µm). ERM formed a less differentiated epithelium. ERM cells grown in 3D organotypic culture did not show any signs of differentiation. The cells forming the epithelium had a basaloid appearance throughout the whole epithelial thickness, in contrast to the epithelium formed by the cells isolated form NOM, that showed a distinct basal cell layer and upper, more differentiated cell

**Fig. 8:**
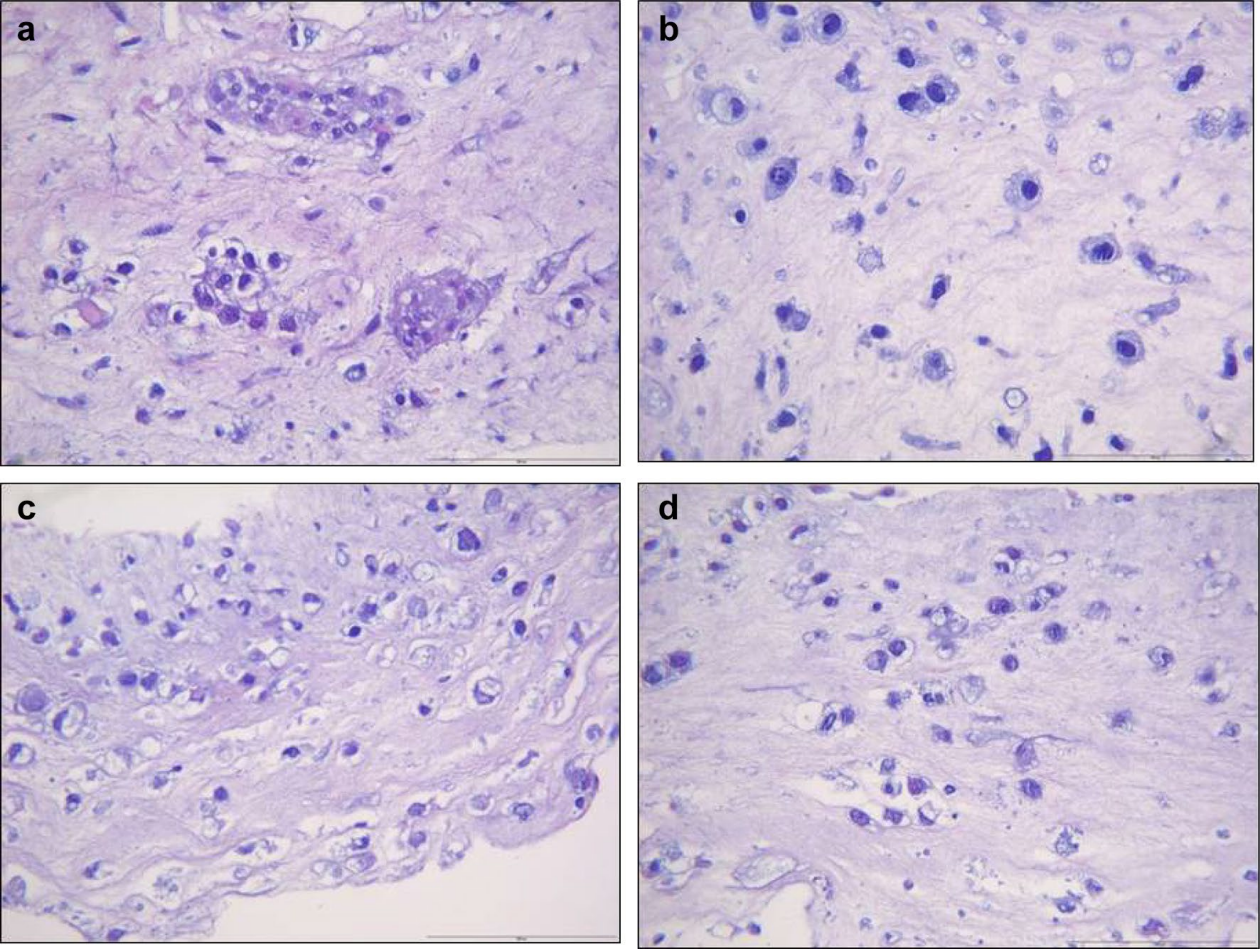
NOM and ERM cells grown in collagen gels. **a** NOM. **b** REM-C. **c** REM-M. **d** REM-A (original magnification × 200, scale bar 100 µm). When grown in 3D, but imbedded within collagen gels and not on top of the collagen gels, the NOM cells formed small islands with central differentiation. ERM cells did not agglomerate; the ERM cells grew alone, as individual cells, detached from each other

When grown in 3D organotypic cultures (Fig. 7) and in collagen gels (Fig. 8), ERM formed a less differentiated epithelium. ERM cells grown in 3D organotypic culture did not show any signs of differentiation. The cells forming the epithelium had a basaloid appearance throughout the whole epithelial thickness, in contrast to the epithelium formed by the cells isolated form NOM, that showed a distinct basal cell layer and upper, more differentiated cell layers.

When grown in 3D, but imbedded within collagen gels and not on top of the collagen gels, the NOM cells formed small islands with central differentiation. ERM cells did not agglomerate; the ERM cells grew alone, as individual cells, detached from each other.

**Fig. 9:**
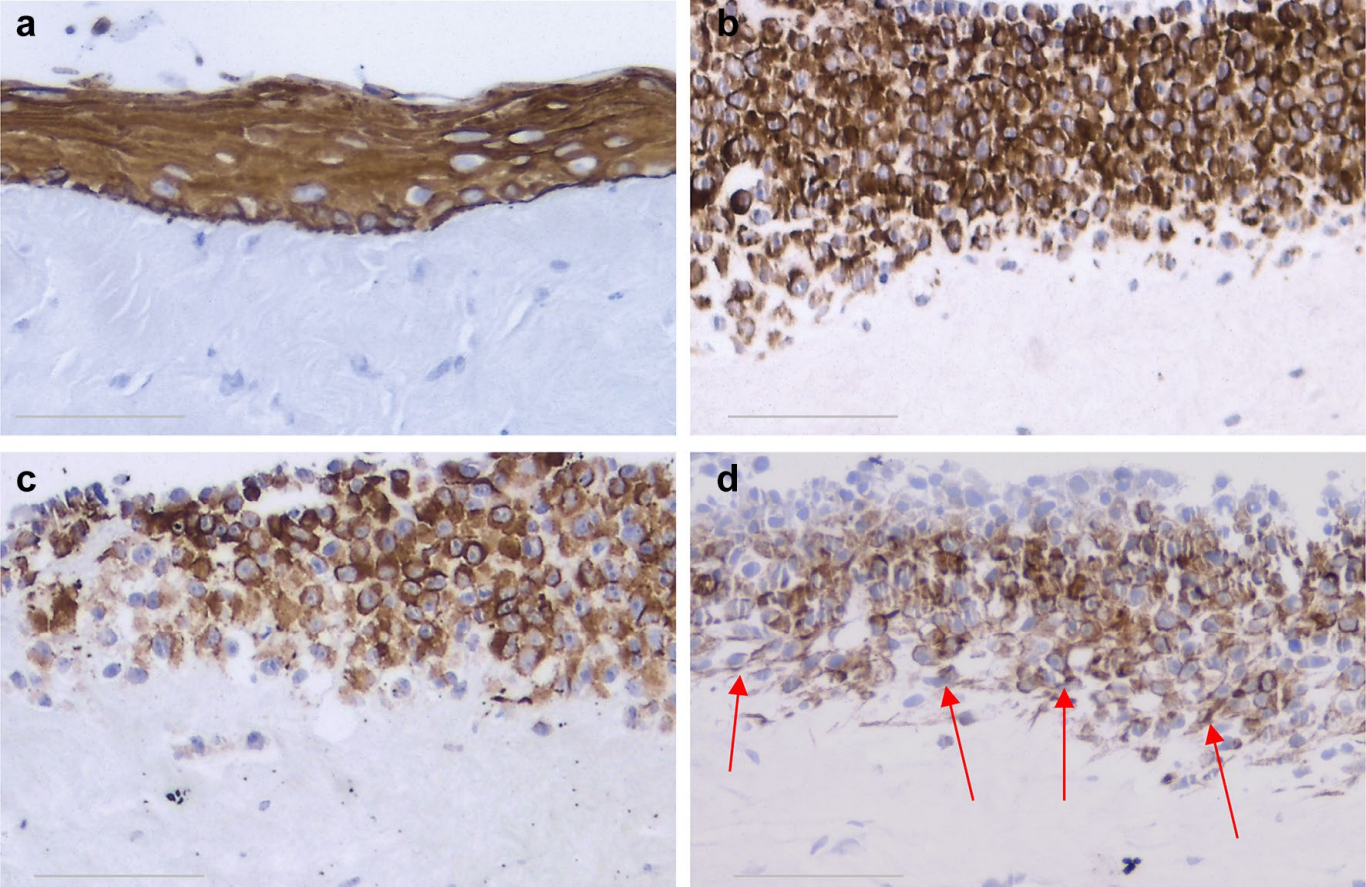
Pancytokeratin staining of NOM and ERM cells grown in 3D organotypic culture. **a** NOM. **b** REM-C. **c** REM-M. **d** REM-A (original magnification × 100, scale bar 50 µm). Epithelium formed by both NOM and ERM cells showed positive staining for pancytokeratin. However, the intensity of the staining gradually decreased from NOM and REM-C till REM-A, which showed the weakest expression of pancytokeratin. Note the pancytokeratin-positive cells in the basal layer of the epithelium formed by REM-A that display an elongated, mesenchymal morphology (arrows)

**Fig. 10:**
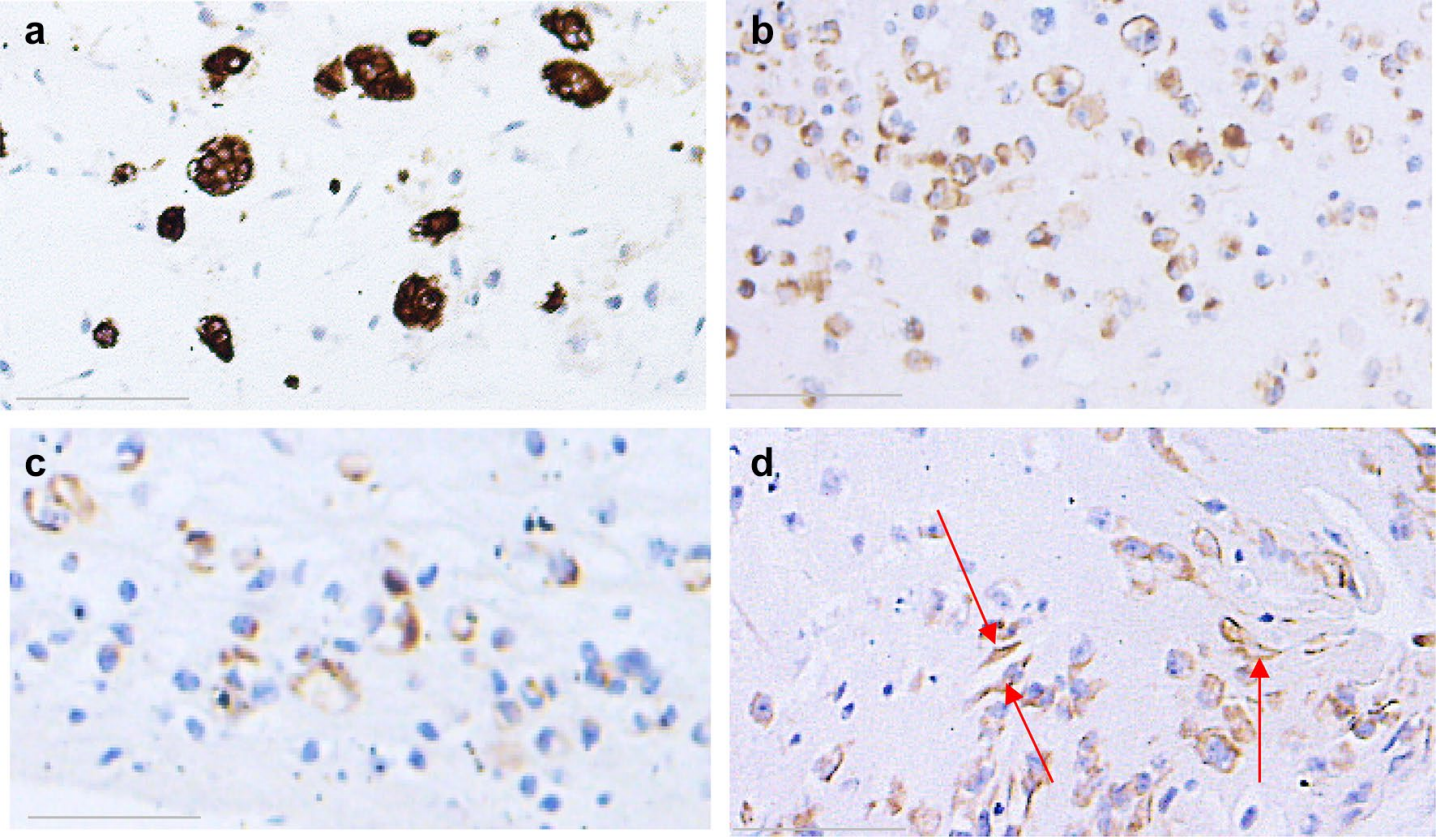
Pancytokeratin staining of NOM and ERM cells grown in collagen gels. **a** NOM. **b** REM-C. **c** REM-M. **d** REM-A (original magnification × 100, scale bar 100 µm). The small islands formed by NOM cells showed intense pancytokeratin staining. ERM cells grown in gels showed also pancytokeratin positivity but much weaker. Note that the REM-A pancytokeratin-positive cells display a mixture of shapes, from rounded, epithelial morphology to elongated, mesenchymal morphology (arrows)

Epithelium formed by both NOM and ERM cells when grown in 3D organotypic cultures showed positive staining for pancytokeratin (Fig. 9). The intensity of the staining gradually decreased from NOM and REM-C till REM-A, which showed the weakest expression of pancytokeratin. Of note, the pancytokeratin-positive cells in the basal layer of the epithelium formed by REM-A in 3D organotypic cultures displayed an elongated, mesenchymal-like morphology. The small islands formed by NOM cells when grown in 3D gels showed also an intense expression of pancytokeratin (Fig. 10). ERM cells grown in gels showed as well pancytokeratin positivity but much weaker. Interestingly, and most predominantly observed in REM-A gels, the pancytokeratin-positive cells displayed a mixture of shapes, from rounded, epithelial morphology to elongated, mesenchymal morphology.

**Fig. 11:**
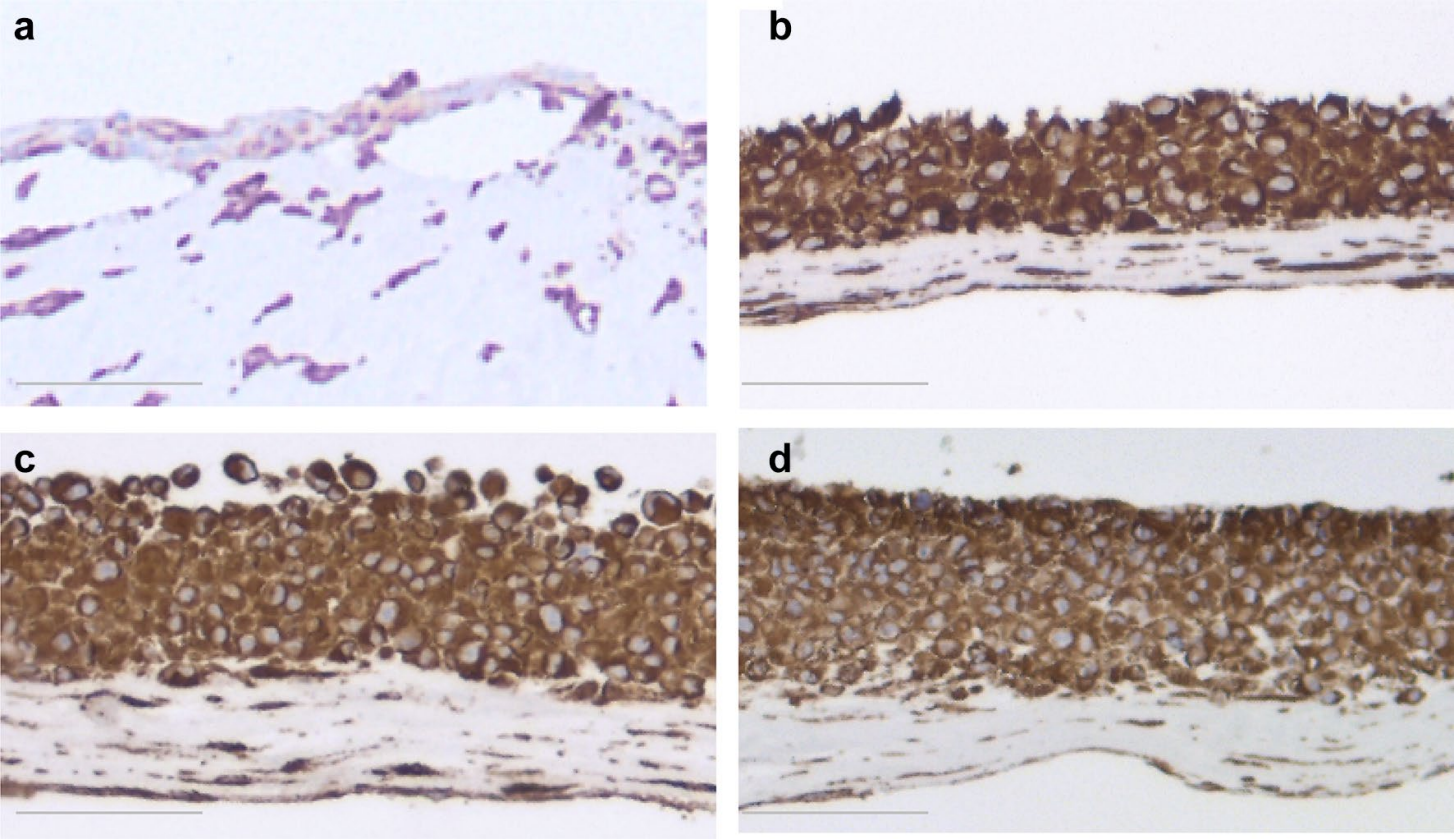
Vimentin staining of NOM and ERM cells grown in 3D organotypic culture. **a** NOM. **b** REM-C. **c** REM-M. **d** REM-A (original magnification × 100, scale bar 50 µm). Epithelium formed by showed scattered positive cells localized mainly to the basal cell layer. Epithelium formed by REM cells showed intense vimentin staining throughout all cell layers

Staining for vimentin showed that epithelium formed by NOM in 3D organotypic cultures showed scattered positive cells localized mainly to the basal cell layer (Fig. 11). Epithelium formed by REM cells showed intense vimentin staining throughout all cell layers, indicating less epithelial differentiation of these cells. All cells showed intense vimentin staining when cultured in gels (data not shown).

## Discussion

Serres [7] first reported that epithelial cell populations existed in PDL tissue. Subsequently, in 1884, Malassez [8] confirmed the existence of epithelial components as well as the idea that these components were the remains of Hertwig’s epithelial sheath within the PDL spread, garnering ERMs much attention.

It has been reported that ERMs are often composed of several cell populations [9, 10] and have elliptical, funicular, or tufted shapes; they may also be reticulate and wrap around the tooth root or form a network with the junctional epithelium [11–14].

Static, proliferative, degenerative, and differentiated states of ERM dynamics have been observed, with some reports also demonstrating division [15, 16].

Ten Cate [17] and Gilhuus-Moe and Kvam [18] reported that these cells proliferate under certain conditions according to radioactive isotopes, electron microscopy, and histological investigations.

It has been reported that ERMs remain near the tooth cervix, bind with the junctional epithelium, and form pocket epithelium [3]. This, along with the idea that infectious antigenic substances resulting from dental pulp diseases are discharged from the root canal through the apical foramen, the proliferation of normally static ERMs is started, thereby causing them to form the lining epithelium of radicular cysts, which is also supported by the fact that ERMs start to proliferate as a result of culture conditions and stimulation [4, 19, 20].

Reported ERM functions include maintaining PDL space width [21, 22], stimulating dental cement formation [23], protecting root resorption [24], controlling ankylosis, tooth instability, and alveolar resorption [25], and being involved in alveolar resorption from marginal periodontitis caused by prostaglandin and non-prostaglandin bone resorption activity factors [26].

Since stem cells were isolated from dental pulp, it has been suggested that post-embryonic stem cells might also exist in the periodontal tissue of human adults [27]. However, the presence of stem cells in PDL received a large amount of attention in 2004 after Seo et al. [28] reported multipotent cells in human PDL. CD44 is an adhesive molecule that binds with extracellular matrices such as hyaluronic acid and is strongly involved in lymphocyte homing, lymphocyte activation, cell-to-cell adhesion, cell-to-matrix adhesion, and cell movement, as well as cancer cell proliferation and metastasis. Thus, it has also been acknowledged as a stem cell marker for various types of solid cancers. In normal tissue, CD44 is distributed throughout various cell lines, including hematopoietic cells, fibroblasts, epithelial cells, vascular endothelial cells, muscle cells, and neuroglial cells and is either expressed or absent in the differentiation and proliferation of each of these cell lines. In squamous epithelial mucosa, CD44 expression is enhanced at the base where proliferation is strong, while it is weakly expressed or not present on the surface areas [29], Moreover, it may be related to the differentiation and proliferation of hematopoietic stem cells and B cells in the dental pulp [30, 31]. Thus, it appears that CD44 is involved in morphogenesis, wound healing, and tumor progression as an extracellular matrix for cell movement. It is noteworthy that the results of FACS analysis revealed that the CD44-positive cell ratio was much higher in cells obtained from PDL than in the oral mucosa.

When grown in 3D organotypic cultures and in collagen gels the cells derived from PDL formed a less differentiated epithelium that expressed weaker pancytokeratin and stronger vimentin, indicating that these cells have a less epithelial phenotype and a more mesenchymal phenotype. That might indicate that those cells are less differentiated and more EMT than the cells derived from NOM. Accordingly, this might be the reason that they did not form a well-differentiated and keratinized epithelium in 3D organotypic cultures, such as the epithelium formed by the cells derived from NOM.

When grown inside the gels, the cells derived from NOM grew more clustered, in groups, while the epithelial cells derived from REM grew alone in the matrix. This again might indicate that the cells from NOM are probably more differentiated and express more epithelial cell-to-cell adhesion molecules, while cells derived from ERM are less differentiated.

Our results are in line with the results published in 2016 by Tsunematsu et al. [32]. They have isolated odontogenic epithelial cells with epithelial marker-positive and mesenchymal marker-negative features from ERMs in human PDL and reported that they have stem cell-like characteristics. The findings we present here bring new information about the extent of the stemness of the differentiation abilities of the cells derived from ERMs compared to the epithelial cells derived from NOM.

We anticipate that the cell groups isolated here will be investigated in more detail in the future for their possible involvement in cyst formation, by developing an experimental model for radicular cyst formation. This model will also provide a valuable experimental biological system for testing of novel, alternative ways of treatment for radicular cysts.

## Conclusions

Epithelial cells could be isolated from the REM existent in adult human periodontium and grown in culture. Their in vitro characterization indicates that cells derived from ERM have a less differentiated phenotype compared with cells derived from normal oral epithelium.
